# Effective bio-activities of the metal nanoparticles mycosynthesized by the plants’ fungal endophytes

**DOI:** 10.1186/s12934-026-03119-2

**Published:** 2026-10-09

**Authors:** Adel K. Madbouly, Younes M. Rashad

**Affiliations:** 1 Microbiology Department, Faculty of Science, University of Ain Shams, Cairo, Egypt; 2 Plant Protection and Biomolecular Diagnosis Department, Arid Lands Cultivation Research Institute (ALCRI), City of Scientific Research and Technological Applications (SRTA-city), New Borg El-Arab City, Egypt

**Keywords:** Green synthesis, Endophytic fungi, Nanotechnology, Antimicrobials, Anticancer activity, Sustainable agriculture

## Abstract

Fungal endophytes act as bio-nano factories and are favored over bacteria and plant extracts, as they secrete large quantities of proteins and enzymes, facilitating stable, efficient, and economic nanoparticle synthesis. This review highlights the advantages of plant-associated fungal endophytes as bio-factories for metal nanoparticles (MNPs) synthesis and critically examines the biological activities and potential applications of these MNPs in human health, food technology, and agriculture. Compared to chemical synthesis, biogenic MNPs are more resistant to aggregation due to the involvement of different biomolecules, including secondary metabolites, enzymes, and proteins, reducing the metal ions to form MNPs. Biosynthesis of MNPs by several fungal genera, such as *Fusarium* spp., *Penicillium* spp., and *Aspergillus* spp., enables alleviation of the undesirable cytotoxicity and increased stability. In the food industry, MNPs play substantial roles in inhibiting browning and preventing food contamination. In agriculture, they act as herbicides, fungicides, pesticides, and plant growth promoters. Field application trials of these MNPs are recommended before their manipulation as a control strategy. In medicine, MNPs behave as antimicrobials, drug-delivery, antioxidants, and anticancer agents. They are applied in ophthalmology to facilitate drug delivery onto the site of infection *via* the improvement of targeted delivery. MNPs have demonstrated anticancer potency against cancer cell lines; however, they may also display cytotoxicity against the normal cells, especially at small sizes. However, at low pH, release of Ag^+^‏ ions from AgNPs increases, where such information may aid in reducing the toxicity of the AgNPs. Conjugation of MNPs and antimicrobials has shown synergistic efficacy against the microbial resistance compared to the antimicrobials alone, reducing the development of antimicrobial resistance and decreasing the duration and the dose requirements of antimicrobial treatments. Nevertheless, comprehensive toxicological, pharmacokinetic, environmental, field, and *in vivo* studies are required before widespread application. The novelty of this review lies in spotting the light on the useful role of fungal endophytes as bio-factories for MNPs biosynthesis and emphasizing the promising applications of these MNPs in human health, food technology, and sustainable agricultural disease management, while highlighting the challenges and research priorities required for their safe and effective translation from laboratory studies to practical applications.

## Introduction

Nanoscale materials can be synthesized through physical and chemical approaches [1] or *via* biological processes mediated by various microorganisms, including filamentous fungi, yeasts, and bacteria [2]. The biogenic methods used for the synthesis of metal nanoparticles (MNPs) are characterized by being simple, low-cost, and ecofriendly alternatives compared to the other methods, which require the use of toxic chemical reagents, high use of energy, and complex instrumentation [3, 4].

Compared to the chemical and physical synthesis approaches, which rely on toxic chemicals and high energy consumption, respectively, green synthesis of NPs using seaweeds such as macro- and microalgae, plant extracts, or microorganisms (*i.e.*, fungi and bacteria) as stabilizing, reducing, precipitating, and chelating agents of metal ions confers bioactive potentials and minimizes the deleterious environmental impact [5]. Furthermore, obtaining MNPs by biological means reduces the chemical pollution’s risk, as no toxic residues will be generated during the biosynthesis. However, it is better to study the risks of the release of these MNPs into the environment, considering any changes that may arise in the environment, which can change their toxicity and properties [6].

Filamentous fungi are promising candidates for MNPs synthesis on industrial scale because of the simplicity and low costs of their cultures [7]. Several fungal genera, including *Fusarium* spp., *Penicillium* spp., *Aspergillus* spp., and *Trichoderma* spp., are commonly used in the bioreduction of metal ions into MNPs form [7]. Endophytic fungi are considered treasure houses of bioactive compounds, mainly flavonoids, terpenoids, carotenoids, and polyphenols [8]. These biomolecules carry out different functions in the endophytes; thus, they may be employed in stabilizing and reducing the metal ions [9]. Utilization of endophytic fungi isolated from potent medicinal plants represents a novel strategy for the production of many metal oxide NPs in a cost-effective and green manner [10]. Endophytic fungi show considerable potential for the biosynthesis of MNPs, with promising applications in promoting ecosystem sustainability and enhancing crop productivity. The practical application of endophytic fungi in agriculture is promising, with demonstrated potential for improving crop growth, productivity, and stress tolerance [11]. Compared to the other sources, endophytic fungi in particular have several advantages for the mycosynthesis of MNPs, including growth in minimal media and extensive extracellular production of enzymes [12]. Recently, the fungal-mediated nanostructures have gained considerable attention due to being ecofriendly, efficient, sustainable, and cost-effective in nature [13].

Nanomaterials possess versatile and unique physicochemical characteristics, which are important for their technological development and application in many fields, including pharmaceutics, treatment of water, the food industry, and agriculture [14]. Nanotechnology is widely used in various agricultural applications, including improvement of plant tolerance to stress through nanozyme-mediated scavenging of reactive oxygen species (ROS) [2], increase of plant tolerance to several environmental stresses such as salinity [15], drought [16], heat [17], and coldness [18], and enabling a controlled and a targeted release of the agrochemicals [19]. Currently, MNP’s study is a topic of remarkable interest, owing to their various ways of application in the biological fields [20]. The objectives of the present review are exploring the potent roles of endophytic fungi in the biosynthesis of MNPs and providing a comprehensive insight on MNPs, such as their nature, types, activities, and recent applications in various fields.

### What are metal nanoparticles?

Nanostructured materials have got noticeable attention due to their fascinating, unique, and size-dependent physicochemical properties, along with their potential applications [21, 22]. Among the various nanostructured materials, noble MNPs, including gold (Au), palladium (Pd), platinum (Pt), and silver (Ag) have attracted considerable interest because of their distinctive optical, catalytic, and physicochemical properties. These properties, which differ from or are enhanced relative to their bulk counterparts, contribute to their broad applications in biomedical, environmental, and agricultural fields [23, 24]. AgNPs are particularly important due to their diverse and surprising applications in various fields, such as drug delivery [25], medical and pharmaceutical fields [26], photothermal therapy [27], catalysis [28], sensing [29], energy conversion [30], optoelectronics [31], and solar cells [32], in addition to their wide ecological applications. Furthermore, MNPs are widely used as they have many advantages, including their high diversity, surface electronic charge, and desirable size and surface-volume ratio [33]. Among these MNPs, AgNPs are outstanding candidates due to their potent bioactivities, mainly anti-inflammatory, antifungal, antibacterial, antiviral, and anticancer, along with their low chemical reactivity and high stability [34].

Inorganic nanostructures are synthesized *via* the precipitation of inorganic substances comprising metal elements, metal salts, and metal oxides (*i.e.*, Fe_3_O_4_, TiO_2_, Al_2_O_3_, MnO, CeO_2_, and ZrO_2_). These inorganic MNPs have been widely investigated for their clinical applications as therapeutic agents, imaging agents, and antiseptics. In the food industry, they have also been explored for inhibiting enzymatic browning, enhancing food biofortification, facilitating food processing and pathogen detection, developing biodegradable food-packaging materials, and preventing food contamination [35].

In the agricultural sector, the obtained findings of Jana et al. [20] revealed that AgNPs may be suitable for formulation as novel antifungals to control the fungal infections of plants, especially in the horticultural crops. Meanwhile, in the medical sector, the safer way to administer a drug into the human body is *via* the use of the small and high loading capacity of MNPs [36]. Conventional eye drops are among the most commonly used approaches for ocular drug delivery and the treatment of eye infections. However, their therapeutic efficacy and ocular bioavailability are often limited by short precorneal residence times and rapid tear turnover, which promote drug drainage and clearance from the ocular surface [37]. MNPs possess unique physicochemical and bioactive properties that may help in improving targeted drug delivery to sites of infection and improve therapeutic efficacy [38]. Furthermore, MNPs have emerged as promising candidates for the development of novel therapeutic approaches to the ocular infections [39].

On the other hand, organic nanostructures can be incorporated into biodegradable polymers to produce bio-nanocomposites with enhanced biodegradability and potential environmental advantages. These organic NPs are compatible with the low-toxicity biological systems, including stable lipids, liposomes, micelles, and polymeric compounds [40].

Engineered nanostructures are produced and released into the environment by human beings [41]. They enhance cation exchange, have beneficial impacts on crop yield, and improve the efficacy of plant disease management strategies. The major characteristics that may influence the toxicity of these engineered nanostructures involve their shape, size, solubility, surface functionalization, aspect ratio, and propensity to agglomerate, along with their binding with the various receptors and proteins [35]. The application of engineered nanostructures in biomedical research has attracted considerable attention because of their chemical stability, biocompatibility, ease of functionalization, and high saturation magnetization that enhances their stability. These properties make them promising candidates for various biomedical applications. However, following their uptake into the human body, some nanostructures may induce the intracellular generation of ROS inside the cells, which can disrupt cellular signaling and promote the oxidation of proteins, lipids, and other biomolecules [35]. Meanwhile, comparative investigations targeting the impacts of various natural compounds on the MNP morphology, bioactivity, and stability are needed to provide valuable insights into the rational design of biogenic AgNPs with optimized therapeutic efficacy. In addition, integrating the theoretical and experimental methods may contribute to the establishment of structure–activity relationships, which explain how the natural product’s molecules can influence the MNPs behavior [42].

### Endophytic fungi

Endophytic fungi are microorganisms that reside within the plant tissues without causing apparent harm to their host plants. They can establish mutualistic associations with their host plants and enhance host resistance to several abiotic stresses such as heavy metal toxicity [43]. Recently, endophytic fungi have attracted considerable research interests because they act as biostimulants for essential oil biosynthesis [44], represent promising resources of several cytotoxic compounds, including molecules with potential anticancer activity [45] and act as sources of antibacterial substances [46]. Furthermore, they may act as biocontrol agents [47], promote plant growth [48], enhance nutrient solubilization in the plant rhizosphere [49], and activate the plant’s systemic resistance to the various abiotic [50] and biotic [51] stresses. In the environment, the increment of heavy metals (HMs) has created an urgent need for an efficient and cost-effective remediation approaches. The study reported by Ponce-Hernandez et al. [52] assessed the efficacy of endophytic fungi isolated from *Typha latifolia* roots to accumulate and tolerate HMs, inhibit phytopathogenic fungi, and promote plant growth. The obtained results of several confrontation assays displayed that endophytic *Talaromyces* spp. inhibited *Fusarium* spp. and *Botrytis cinerea*, where all tested fungal isolates expressed a chitinolytic activity. The findings of this study revealed that endophytic *Talaromyces* fungi acted as potent biofertilizers and biocontrol agents against several phytopathogenic fungi, and displayed a considerable potential for bioremediation of HMs-contaminated aquatic sites, thereby enhancing the plant bioremediation ability. Due to its soil persistence and race-specific virulence, *F. oxysporum* f. sp. *lycopersici* is a highly destructive fungal vascular pathogen, which causes a remarkable global yield losses. The use of an ambitious and ecofriendly strategy to controlling Fusarium wilt, involves the use of endophytic fungi, especially those that are plant growth-promoting ones. These fungi colonize plant tissues without causing apparent harm and can improve its growth, nutrient uptake, and tolerance to various biotic and abiotic stresses through multiple direct mechanisms, including competition for nutrients and space, mycoparasitism, and antibiosis, along with several indirect mechanisms such as microbiome modulation, hormonal regulation, and induction of systemic resistance. Many endofungal species have shown a substantial antifungal potential, growth-promoting effects, and the ability to enhance antioxidant defenses in various. host plants [53].

Medicinal plants are recognized as storehouses of endophytic fungi that are able to synthesize several important novel pharmaceutical secondary metabolites (SMs) [54]. Moreover, these endophytic fungi are regarded as efficient sources of various biologically active phytohormones, which support the plant to grow and make their hosts able to survive in the presence of various stresses [55]. Commercially, some of the most important usable SMs that are biosynthesized by the endophytic fungi include the anticarcinogenics, antibiotics, allelopathic compounds, insecticides, and cytotoxics [56]. Endiofungi are recognized as metabolically more active than their free counterparts, so they are considered as resources of novel metabolites with potential bio-applications in several fields, such as agriculture, medicine, and the food industry [57]. Furthermore, the Zheng et al. [58] study has reported that endophytic fungi have substantial biotechnological activities, mainly biotransformation, enzyme production, and bioremediation.

Fungal endophytes have emerged as eco-friendly alternatives to the conventional chemical approaches used for the synthesis of MNP’s. A previous study carried out by Birla et al. [59] revealed that the *Helminthosporium* sp. and *Chaetomium* sp. filtrates have been subjected to the treatment with AgNO_3_, where the reaction mixture has exhibited a change from colorless to brown color, indicating the formation of AgNPs. Abdel-Hafez et al. [60] attributed this change in color to the reduction of Ag^+^ ions into AgNPs. These AgNPs have expressed a recognizable antifungal potency against several fungal phytopathogens, including *H. maydis*, *Phytophthora infestans*, and *B. cinerea*. Interestingly, AgNPs treatment of cherry tomatoes has effectively suppressed the *B. cinerea* growth and minimized the fruit’s weight loss. These findings underscore the effectiveness of the biosynthesized AgNPs *via* acting as disease control agents, representing promising treatments against fungal infections of the postharvest crops [61]. In accordance, in a recent study conducted by Mostafazade et al. [62], AgNPs biosynthesized by several *Fusarium* spp. (*i.e.*, *F. avenaceum*, *F. falciforme*, and *F. oxysporum*) endophytically associated with an endemic plant of Iran (*Salvia persepolitana*) have been regarded as a potent alternative to the traditional chemical control strategies.

### Mycosynthesis of metal nanoparticles

Green synthesis of MNPs involves the use of several biological systems, including plants, algae, bacteria, and fungi, as bio-fabrication platforms [63]. This approach has emerged as a cost-effective, ambitious, and eco-friendly alternative to the traditional physical and chemical methods. MNPs provide a substantial biomedical potency, attributed to their multi-functionality, stability, and biocompatibility [64].

Compared to the other microorganisms, fungi are considered promising candidates for the biosynthesis of MNPs because of their relative safety, high biosynthetic capacity, and several practical advantages. They are characterized by a high bioaccumulation ability, metal tolerance, rapid growth rate, efficient scale-up mechanisms, higher productivity, and ease of handling [35]. Many of these fungi are resistant to the various metals and are able to bioaccumulate the metal ions in their spores and mycelia [65]. Additionally, compared to the other microorganisms, fungi secrete a diverse array of enzymes and SMs and can produce high yields of proteins. Their relatively low cost, ease of cultivation and manipulation, and efficient extracellular biosynthetic capabilities further support their use as promising platforms for MNP synthesis [1]. Regarding the scale-up production processes, the mold fungi can withstand flow pressure, agitation, and other necessary conditions. A previous study reported by Vetchinkina et al. [66] demonstrated that compared to the chemical MNPs, the biogenic MNPs display more tolerance to aggregation, due to the involvement of different biomolecules during the reduction process, such as proteins, SMs, enzymes, and stabilizing and capping agents. Mycosynthesis of AgNPs enables alleviation of the undesirable cytotoxicity, increased stability, and simultaneous capping with the bio-agents [67].

Biosynthesis of MNPs involves the manipulation of several microorganisms, including mold fungi, yeasts, and bacteria, in addition to plant extracts. Manipulation of fungi is more advantageous than that of bacteria, as they possess a good biomass, ease of recovery, and do not necessitate additional steps for the fungal filtrate extraction [68]. Srivastava and Bhargava [35] reported that fungi are more effective compared to bacteria, especially at low pH, due to their biosorption of metal ions. Furthermore, Adeleke et al. [11] reported that for MNPs production, fungi are favored over the other microorganisms due to the manageable nature of their biomass. A previous study revealed the conductivity, catalytic efficiency, and chemical stability of the MNPs synthesized by the endophytic fungi [69].

Myconanotechnology is an emerging and rapidly expanding field of applied science, which has gained the attention of several biomedical researchers [70]. The mycosynthesis of AgNPs represents an eco-friendly innovation that offers sustainable biosynthetic approaches, environmental benefits, and potentially cost-effective processing [71]. Recently, utilization of endophytic fungi as nanofactories for mycosynthesis of MNPs has become increasingly more common worldwide [72]. Mycogenesis of nanostructures relies on several parameters, such as temperature, pH, concentration of the microbial biomass, type of MNPs precursor, and time of incubation [7]. Fungal strains, substrates, and culture conditions act as important determinants of the MNPs biosynthesis *via* several modes of action, entailing a complex interaction of several elements [11]. The extensive surface area of endophytic fungal mycelia, simple and inexpensive culture media, well scalability, and ability to secrete various enzymes make them suitable representatives for the production of MNPs *in vitro* and on an industrial scale [42, 73].

The fungal-mediated biosynthesis of the MNPs proceeds *in vitro* and *in vivo*. *In vivo* biosynthesis, or the intracellular method, takes place primarily inside the living fungal cell [74]. The fungal cells reduce the metal ions to form MNPs because they are toxic to these cells, which is considered a type of defense mechanism against the toxic substances. One limitation of this synthesis approach is that the resulting MNPs must be extracted from the fungal mycelial biomass before downstream applications. On the other hand, *in vitro* biosynthesis of the MNPs encompasses three different pathways, which do not require extraction from the cells of fungi. The first strategy utilizes fungal culture supernatants, which include proteins, enzymes, and other components secreted extracellularly into the growth medium. The second strategy employs extracts of the fungal post-culture, involving several intracellular compounds that have the ability to biosynthesize the MNPs. Finally, the third strategy uses an aqueous solution that is obtained after incubation of the fungal culture in water [68]. As an advantage of MNPs mycogenesis, the extracellular secretion of proteins and other SMs by the fungal biomass is notably higher. Precipitation of the biogenic MNPs synthesized by the fungal biomass outside the cell enables their utilization in the diverse areas [35]. Furthermore, Zhao et al. [75] revealed that the extracellular methods may produce a much wider range of MNP’s sizes (*i.e.*, 1–200 nm), compared to the intracellular counterparts (25–60 nm). Although marine endophytic fungi have been studied for their diverse medicinal properties, such as anticancer activity, their potential for the bioreduction of Au ions and the biosynthesis of AuNPs remains largely unexplored. Interestingly, the seaweed endophytic fungi exist in special environmental niches, making them suitable producers of novel therapeutic MNPs [76].

Mycosynthesized MNPs have shown promising antimicrobial activity against a broad range of microorganisms while generally exhibiting lower toxicity towards mammalian cells; however, their biosafety depends on their composition, physicochemical properties, concentration, and exposure conditions [77]. However, some filamentous fungi used for extracellular biosynthesis of AgNPs, including *A. fumigatus* and *F. oxysporum* are known to be pathogenic to humans and/or plants, which may raise biosafety concerns regarding their use in NPs production [78]. Accordingly, successful biosynthesis of AgNPs requires the development of mycosynthetic approaches that utilize the non-pathogenic fungal species. de Oliveira et al.‘s [79] highlighted the importance of developing new synthesis strategies of the MNPs that may integrate mycosynthesis with sustainability, providing a profound alternative for the biosynthesis of environmentally safe nanomaterials, which have beneficial bio-applications in the agricultural and the human health sectors.

Fungal endophytes represent promising biological nanofactories because they offer a combination of cost-effective processing, high-yield production, ecofriendly scalability, and enhanced stability of the resulting MNPs. Unlike non-endophytic fungi, the endophytic fungi produce a diverse array of bioactive metabolites, including phenols, alkaloids, and terpenoids, thereby acting as effective capping and stabilizing agents, resulting in the production of highly functional and less agglomerated MNPs. Furthermore, they provide a higher yield of proteins and are simpler to treat *in vitro* [80]. The unique cellular organization and metabolic capabilities of filamentous fungi have attracted considerable interest in endophytic fungal-mediated synthesis of AgNPs as an alternative to nanoparticle biosynthesis using other microorganisms [81]. The eco-friendly nature, relatively low toxicity, and cost-effectiveness of endophytic fungal-mediated AgNP synthesis have increased interest in the development of nanoparticle-based therapeutics and mycofabricated AgNPs [69]. Gaikwad et al. [82] successfully synthesized AgNPs through a green approach using six different *Fusarium* species, including *F. solani*, *F. graminearum*, *F. oxysporum*, *F. scirpi*, *F. tricinctum*, and *F. culmorum*. The size of these mycosynthesized AgNPs lies in the range of 24–45 nm. The obtained results revealed that *F. oxysporum* produced the smallest-sized AgNPs, whereas *F. tricinctum* produced the largest AgNPs. In another study, *A. fumigatus* was successfully employed for the synthesis of AgNPs and multi-walled carbon nanotubes in a catalytic system. The electron microscopy (EM) analysis revealed well-dispersed spherical AgNPs with an average diameter of approximately 10 nm deposited on the carbon nanotubes [35].

Overall, endophytic fungal-mediated synthesis of MNPs represents a promising green approach that can reduce the generation of hazardous wastes compared to conventional chemical synthesis, which often relies on potentially toxic reducing and stabilizing agents. The endofungal enzymes, SMs, and proteins act as reducing agents (converting metal ions to MNPs) and capping agents (stabilizing the MNPs); thus, reducing the need for synthetic stabilizers. Optimization of the cultivation conditions such as temperature and pH permits the production of homogeneous MNPs with promising sizes and specific shapes. The fungal endophytes offer rapid growth rates in the traditional laboratory media and produce remarkable MNPs.

### Mechanisms of metal nanoparticles mycosynthesis

**Fig. 1:**
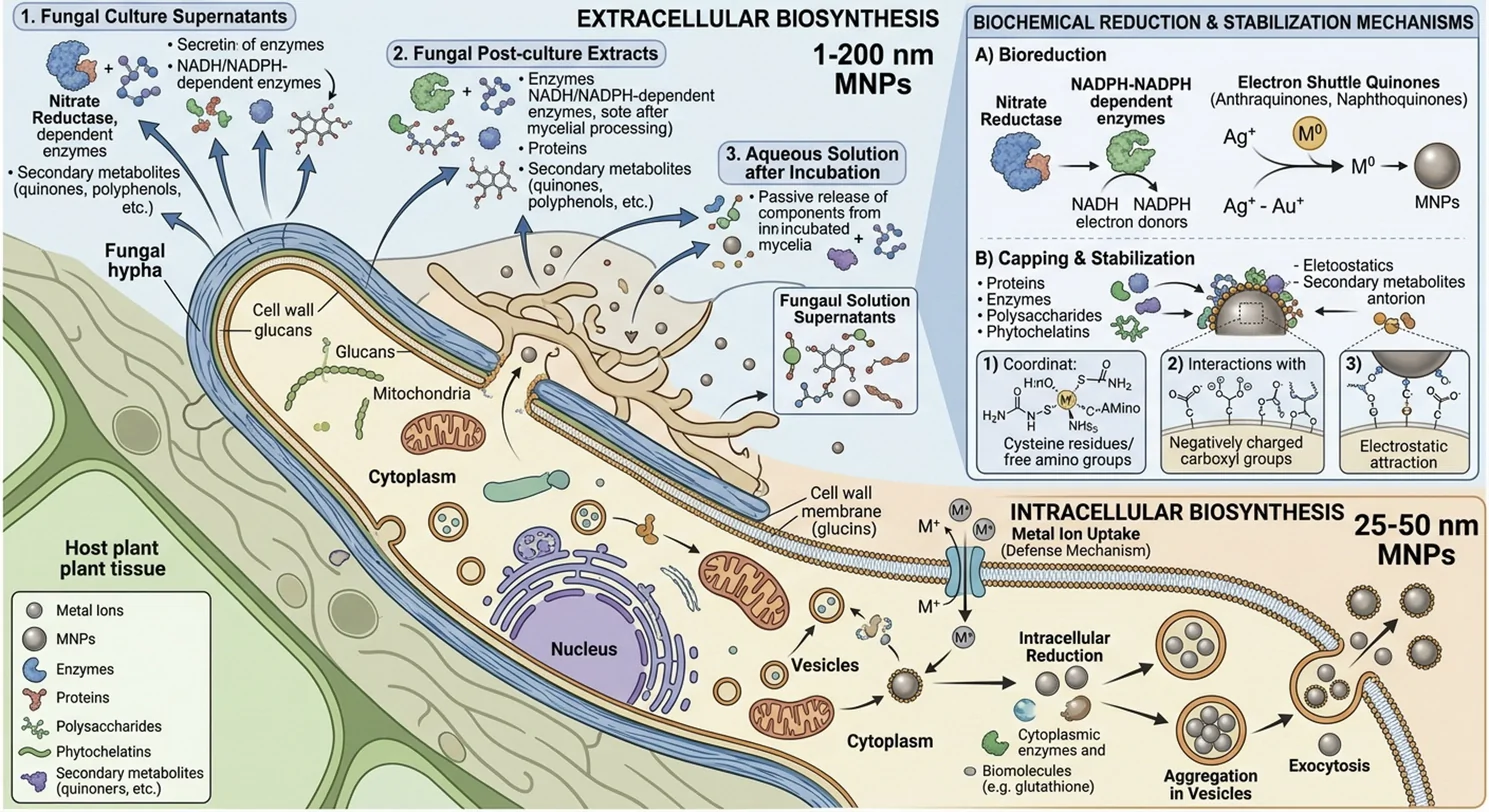
Extracellular and intracellular pathways and biochemical mechanisms of endophytic fungal-mediated metal nanoparticle biosynthesis, highlighting enzymatic bio-reduction (nitrate reductase, electron shuttles) and functional capping/stabilization by fungal biomolecules

Fungal endophytes employ several mechanisms for the MNPs biosynthesis. A previous study reported the MNPs biosynthesis by endophytic fungi through the production of biomolecules such as SMs and enzymes, which are used in the bio-reduction of Ag^+^ ions to form AgNPs [83]. During biosynthesis, the presence of several enzymes such as nitrate reductase and NADH-dependent enzymes is necessary, as they are responsible for enzyme-mediated reduction of metal ions. Jain et al. [84] reported that extracellular fungal SMs, including terpenes, polyphenols, alkaloids, polyketides, and quinones, play major roles in the biosynthesis of AgNPs. Although the biosynthesis of MNPs looks simple, several experimental parameters, such as temperature, agitation, reaction time, and light, need proper optimization to achieve stability, monodispersion, and biocompatibility of the formed MNPs [35]. Furthermore, large quantities of extracellular proteins are produced by several fungal species, conferring stability to the biosynthesized MNPs [35]. The “secretome” encompasses all proteins secreted by a fungus into the surrounding medium. Several enzymes and metal-binding biomolecules such as glutathione reductase (FAD-dependent), α-NADPH-dependent nitrate reductase, phytochelatins, and cytochrome b5 reductase are known to participate in the metal bio-reduction. The biosynthesis of MNPs is often accompanied by the adsorption of biomolecules, including proteins, enzymes, polysaccharides, and other metabolites, onto the nanoparticle surface, where they act as natural capping and stabilizing agents. This surface functionalization helps prevent nanoparticle aggregation and agglomeration and contributes to the colloidal stability of the resulting MNPs. The binding of proteins and other biomolecules to the surface of MNPs contributes to nanoparticle stabilization through interactions involving several functional groups and surface species. These interactions may include (1) adsorption of hydroxide ions onto the surfaces of the MNPs, (2) coordination of metal ions with cysteine residues and free amino groups of proteins, and (3) interactions between the nanoparticle surface and negatively charged carboxyl groups present in fungal cell-wall components and extracellular biomolecules. Zhao et al.‘s [75] reported that fungal cell membrane, cell wall, proteins, and enzymes, along with several organic molecules, exhibit major roles during the biosynthesis of AgNPs. Meanwhile, Ag^+^ ions that form AgNO_3_ become reduced by the enzymes present on the surface of the fungal cell. Additionally, the AgNPs are stabilized by the fungal peptides and proteins through several mechanisms as follows:

1. The biosynthesis of AgNPs may involve electron shuttle quinones and/or the fungal cell wall polymers. The anthraquinones and naphthoquinones present in *F. oxysporum* act as electron shuttles during bio-reduction of the Ag^‏+^ ions [85].

2. Enzyme-mediated reduction: The reduction of Ag‏^+^ ions can be catalyzed by nitrate reductase enzymes [86]. *F*. *oxysporum*, for example, can biosynthesize AgNPs through the activity of NADPH-dependent nitrate reductase, with phytochelatins potentially contributing to nanoparticle capping and stabilization [87]. In addition to fungal enzymes, the anthraquinones and quinine derivatives of naphthoquinones can function as redox-active compounds that facilitate electron transfer during the bio-reduction of Ag⁺ ions [85].

3. Due to the existence of an electrostatic attraction between the cysteine residues and the free amine groups in the fungal proteins and the carboxylate groups, AgNPs are stabilized by the fungal peptides and proteins [88]. Through the action of enzymes in the fungal cell wall, these electrostatic interactions that exist on the fungal cell surface may trap the Ag^+^ ions and reduce them, causing the formation of AgNPs on the fungal mycelia [89]. AgNPs can be biosynthesized both intracellularly and extracellularly by *F. oxysporum*. AgNPs are biosynthesized intracellularly by the reaction of AgNO_3_ with biomass of *F. oxysporum*, where they become accumulated in the cytoplasm and then aggregate finally in the vesicles. Finally, exocytosis can be used to secrete these AgNPs out of the cells. Using this approach, the produced AgNPs were predominantly spherical of 25–50 nm diameter, although larger aggregates measuring approximately 100 nm were also observed [90]. AgNPs can be formed extracellularly by *F. oxysporum* through the action of reducing agents, which are released into the reacting solution. These extracellular AgNPs have diameters in the range of 5–15 nm and become stabilized *via* proteins produced by the *F. oxysporum* in solution [91]. There are diverse MNPs-producing microorganisms, mainly fungi. Fungi are considered the most imperative microorganisms that produce extracellular MNPs due to their remarkable secretory components, which are involved in the capping and reduction of these MNPs [92]. MNPs can be biosynthesized by *T. asperellum* through a process that follows Michaelis–Menten-type kinetics. The mechanism underlying this mycosynthesis process depends on the selective enhancement of the vibrations of certain chemical bonds in the capping agents, possibly a nitrogenous biomacromolecule or proteins [78]. Using a white rot fungus, the proteins may be responsible for stabilizing the AgNPs during their extracellular biosynthesis [93]. In conclusion, the mechanism of intracellular biosynthesis of AgNPs with a fungal biomass differs from that of the extracellular one. In the extracellular mechanism, formation of AgNPs is catalyzed by the action of fungal enzymes released into the reaction solution. However, in the intracellular biosynthesis, MNPs are produced inside the cell cytoplasm, followed by aggregation in vesicles and secretion through the fungal cell membrane by exocytosis [35]. The extracellular and intracellular pathways involved in the biosynthesis of MNPs by endophytic fungi, including enzymatic bio-reduction and stabilization by fungal biomolecules, are illustrated in Fig. 1.

### Bio-activities of the metal nanoparticles

Agriculture is a fundamental sector for human sustenance and socioeconomic development. The global human population is projected to exceed 9.7 billion by 2050 and 11 billion by 2100 [94]. Currently, meeting the global food demand of approximately 7.9 billion people requires the production of around 2–3 billion tons of food grains annually, and this demand is projected to increase by approximately 50% in the coming decades [95].

Due to multiple biotic and/or abiotic stresses, plant diseases may arise [96]. The biotic stressors that induce different forms of plant diseases and cause remarkable loss of agricultural productivity by 20–40% include the pathogenic fungi, bacteria, and viruses, along with pests and weeds [97]. Plant diseases interfere with crop yield and quality; thus, reducing the amount of available food for humans, causing food insecurity and starvation, followed by death [98]. Furthermore, the phytopathogenic fungi produce various mycotoxins, representing one of the harmful effects of fungal plant diseases on humans [99]. Several types of these mycotoxins are immunotoxic, carcinogenic, and nephrotoxic [100].

To combat the deleterious plant diseases, MNPs can be employed as a disease control approach. During the next few years, manipulation of nanotechnology is expected to increase for sustainable agriculture. Manipulation of AgNPs to preserve vegetables and fruits has been investigated recently due to their antimicrobial characteristics [101]. AgNPs application before pathogen exposure prevents infection, providing protection for vegetables and fruits [102]. AgNPs can adhere to fruit surfaces and exert a direct antagonistic effect against invading pathogens [103] and delaying fruit spoilage due to their antioxidant activities [104]. A suitable dosage of AgNPs may stimulate the fruit’s defense mechanisms against pathogens attach; thus, increasing its resilience to infection [61]. The other potential agricultural applications of MNPs involve their use as nanoherbicides [105], nanopesticides [106], nanofertilizers [107], antimicrobial agents [108], promoters of plant growth and seed germination [109], and enhancers of soil function and structural integrity [110], along with stress tolerance [111]. For example, application of AgNPs in agriculture will lead to multiple positive effects on root germination. AgNPs may enhance the seed germination and starch metabolism by combining with the seed coats and aid in the uptake of water inside these seeds. Furthermore, AgNPs are used as nano-priming agents to improve rice-aged seeds and their starch metabolism [112]. Interestingly, before the practical application of the nano-agrochemicals in sustainable agriculture, several challenges must be addressed, including the limited number of field trails, nanoparticle aggregation and stability, size distribution, and control of crystal growth [113]. Finally, MNPs biosynthesized by fungal endophytes may considerably promote the growth of the agricultural crops.

### Antimicrobial potential

Gupta et al. [69] reported that the eco-friendly nature, relatively low toxicity, and low cost of endophytic fungal-mediated biosynthesis of AgNPs have increased interest in the development of nanodrugs and/or mycofabricated AgNPs. With the considerable increment in bacterial drug-resistance and scarce development of novel antibiotics, the biosynthesis of AgNPs is emerging as a safe, suitable, ecofriendly, and broad-spectrum alternative for several biomedical applications. Moreover, a wound healing and anti-biofilm potentials have been observed for these MNPs [114]. Lv et al. [115] reported the isolation of AgNPs that are green synthesized using an aqueous extract of the endophytic fungus *Calonectria eucalypti* (HECL10) recovered from the *Saxifraga stolonifera* plant. Drug susceptibility assays demonstrated that HECL10-AgNPs exhibited a potent antimicrobial efficacy against several pathogens, including *Candida albicans*, *Staphylococcus aureus*, *Pseudomonas aeruginosa*, and *Escherichia coli*.

Moreover, the cytotoxicity assays revealed that HECL10-AgNPs had a remarkable anticancer potential against the HepG2, HeLa, A549, and A375 cell lines. Treatment of HaCaT cell lines with HECL10-AgNPs (25.00 µg/ml) reduced the ROS levels within the cells considerably by 44.8% and promoted the activity of several antioxidant enzymes, mainly glutathione synthetase (GSH), superoxide dismutase (SOD), and catalase (CAT), by 43.5, 106.7, and 2036.1%, respectively. These results demonstrated the promising manipulation of these HECL10-AgNPs in several biomedical applications, such as nanomedicines, in the near future. Multiple mechanisms have been determined that underlay the antimicrobial potential of AgNPs, which are reported as follows [75]:

1. AgNPs attach to the bacterial cell surface and can penetrate the cell membrane. Subsequently, they interact with sulfhydryl groups (-SH) of cellular proteins and enzymes and disrupt membrane lipids and protiens, thereby increasing membrane permeability and causing leakage of intracellular components, including K^+^‏ ions from the cytoplasm. AgNPs have also been suggested to inactivate and bind to the main functional enzymes impairing their normal functions and ultimately leading to cellular damage and lysis [116],

2. AgNPs disrupt bacterial ATP production and interfere with the respiratory chain, leading to excessive formation of ROS and ultimately causing cell death. Proteomic investigations have further demonstrated the upregulation of a panel of envelope-associated and heat-shock proteins, indicating a strong cellular stress response associated with membrane damage and disruption of cellular homeostasis. These proteomic changes, together with the observed impairment of ATP production, provide evidence that AgNPs adversely affect bacterial energy metabolism and membrane integrity [117],

3. The MNPs can interact with bacterial DNA and proteins and potentially interfering with their replication thus, inducing molecular and cellular damage. The observed interactions between the bacterial cells and the MNPs have been attributed to the electrostatic attraction between the positively charged AgNPs and the negatively charged bacterial cell surface [118]. Consequently, mycosynthesized MNPs have emerged as promising nono-based antimicrobial agents exhibiting strong antibacterial, antifungal, and antiviral potentials against antibiotic-resistant microbial strains.

Although MNPs may possess same surface-to-volume ratios, their antimicrobial efficacy is considerably affected by several physicochemical characteristics, particularly particle shape [119] and size [120]. Higher antimicrobial activity is often observed for MNPs with smaller particle size [121]. On the contrary, several reports revealed more effectiveness of the larger MNPs, suggesting that their activity and toxicity do not rely on the size alone [122]. Highlighting the shape-dependent antimicrobial activity of the MNPs, El-Zahry et al. [123] reported that the hexagonal AgNPs with a larger surface area displayed the highest antimicrobial potential compared to the spherical and triangular AgNPs, which was attributed to their larger surface area. The MNP’s charge is another determining factor in managing their antimicrobial activity. The degree of electrostatic interactions between the MNPs and the microbial cell wall may largely control their antimicrobial efficacy. Positively charged MNPs can interact with the negatively charged bacterial cell membrane and disrupt membrane potential and electron transport processes, thereby increasing membrane permeability and causing leakage of intracellular components, ultimately resulting in bacterial cell death [124].

Increasing the MNP’s solubility in the aqueous media may improve their antimicrobial properties. An increase in the solubility of the AgNPs that have been green synthesized using the *Pulicaria glutinosa* aqueous extract is observed upon increasing the concentration of this extract from 4% to 21%. Additionally, the half-maximal inhibitory concentration (IC_50_) of MNPs was lower against *Micrococcus luteus*,* E. coli*, *S. aureus*, and *P*. *aeruginosa* strains [125]. Although MNPs exhibit high antimicrobial efficacy to antagonize the microbial pathogens, their low solubility leads to increased toxicity and a remarkable loss of antimicrobial capacity. Several important physicochemical characteristics of MNPs, including particle size, shape, surface area, surface charge, and solubility, can influence their interactions with microorganisms and, consequently, their antimicrobial activity [126].

The antimicrobial potency of MNPs is affected by their method of synthesis. Garibo et al. [127] reported that AgNPs green-synthesized using *Lysiloma acapulcensis* extract exhibited higher antimicrobial efficacy compared to the chemically synthesized AgNPs. The antimicrobial potential of biogenic AgNPs was evaluated against four tested clinical strains: *C. albicans*, *E. coli*, *P. aeruginosa*, and *S. aureus* using *in vitro* disk diffusion assays, minimal inhibitory concentration (MIC) determination, and minimal bactericidal concentrations (MBC) assays. The obtained results revealed that a larger zone of inhibition induced by the biogenic AgNPs, and lower MIC and MBC have been observed, compared to the chemically synthesized MNPs [127].

### Antibacterial activity

Jiang et al. [128] reported that AgNPs possess different pathways of bactericidal potential. Furthermore, production of ROS or release of Ag^+^ ions from AgNPs may elicit downstream harms to the bacterial cell wall through linkage with the cell’s respiratory enzymes. Dinesh et al. [129] reported the biosynthesis of AgNPs using *P. cinnamopurpureum*, an endophytic fungus isolated from the medicinal plant *Curculigo orchioides*. The mycosynthesized AgNPs exhibited potent antibacterial activity against both Gram-positive and Gram-negative bacteria. The antibacterial activity of AgNPs has been tested against several species of pathogenic bacteria; mainly *Bacillus subtilis*, *S. aureus*, *E. coli*, and *P. aeruginosa*. AgNPs displayed a promising bactericidal activity against these tested pathogens. AgNPs produced inhibition zones ranging from 18 ± 1.66 to 19 ± 1.95 mm. In this study, the *P. cinnamopurpureum* filtrate alone did not exhibit antibacterial activity against the tested bacteria; therefore, the detected antibacterial activity of AgNPs may be attributed to the combined effects of Ag^+^‏ ions and bioactive compounds present in the fungal extract. Another study hypothesized that bacterial inhibition is associated with electrostatic interactions between AgNPs and the bacterial cell surface, which may disrupt the proton motive force and membrane integrity. Consequently, AgNPs can penetrate bacterial cells and interfere with essential cellular processes, ultimately impairing bacterial viability [130]. In a previous study, Jyoti et al. [131] reported that AgNPs (100 µg/ml) mycosynthesized using an endophytic fungus isolated from *Cassia fistula* (a medicinal plant) exhibited considerable antibacterial activity with inhibitory zone diameter (IZD) of 19 ± 0.43 mm against *S. aureus*, 22 ± 0.33 mm against *E. coli*, and 22 ± 0.63 mm against *Klebsiella pneumonia*. The mycosynthesized AgNPs showed a strong antibacterial activity, whereas the AgNO_3_ solution demonstrated no inhibition zone against the tested bacteria. An IZD produced by AgNPs (100 µg/ ml) was greater against *E. coli* than that produced by streptomycin, whereas similar IZDs were observed for *K. pneumoniae* and *S. aureus*. This bactericidal activity was attributed to the existence of bioactive constituents (*i.e.*, amino acids and proteins) on the surface of AgNPs. This characteristic enhanced their antibacterial efficacy by promoting the formation of irreversible pores in the bacterial cell membrane, leading to membrane disruption, leakage of intracellular components, and ultimately bacterial lysis and cell death [132]. AgNPs possess a smaller size and thus interact more closely with the bacterial cells. According to Jyoti and Singh [133], AgNPs exasperate the penetrability and respiratory functions of the bacterial cells by binding to the sulphur- and phosphorus-containing compounds, including the DNA existing in the bacterial cell membrane, causing cell death.

According to Mostafazade et al. [62], the agar well diffusion assay results revealed that as the concentration of the mycosynthesized MNPs has increased, their *in vitro* antibacterial efficacy has shown a remarkable increment against *S. aureus* and *E. coli*, indicating a clear dose-dependent relationship. Moreover, at a fixed concentration of the MNPs, they displayed a greater activity against *S. aureus* compared to *E. coli*. This was attributed to the complexity of the Gram-negative bacterial cell wall structure and the presence of lipopolysaccharide, making it difficult for MNPs to penetrate the cells [134]. Meanwhile, these MNPs also exhibited considerable antibacterial activity against Gram-negative bacteria, which possess a thinner peptidoglycan layer than Gram-positive bacteria, potentially facilitating nanoparticle interaction with and disruption of the bacterial cell envelope. The interaction of the MNPs with the bacterial cell wall, and their final damage to the cell membrane is considered the major mode of their observed antibacterial efficacy [135].

Bagur et al. [136] evaluated the antibacterial efficacy of biogenic AgNPs and an endophytic fungal extract (EFE) against several Gram positive (*S. aureus*) and Gram negative (*E. coli*, *P. aeruginosa*, and *K. pneumonia*) bacteria using *in vitro *disk diffusion, time-kill, and quantitative MIC assays. The disk diffusion assay confirmed the presence of an increment in the antibacterial activity of the biosynthesised AgNPs compared to the EFE, where clear zones of inhibition were observed around the discs loaded with the AgNPs and the EFE. This effect may be attributed to the combined activity of the released Ag^+^ ions and the bioactive compounds derived from the EFE and associated with the nanoparticle surface. In another study conducted by Tripathi et al. [137], the observed remarkable antibacterial activity of the biogenic AgNPs tested against the Gram-positive and the Gram-negative bacteria may be attributed to the synergy between the Ag^+^ ions released from AgNPs in addition to the activity of several bioactive SMs derived from the EFE. Verma et al. [138] reported that the corona formed by active SMs from the EFE on the surface of MNPs may enhance their dispersibility in an aqueous suspension. This indicates a further advantage of a promoted bioavailability; accordingly, a better therapeutic activity. Ali et al. [139] reported the mycosynthesis of AgNPs using *A. parasiticus*, a fungal endophyte isolated from *Reseda Arabica* leaves, and evaluated their antibacterial activity against methicillin-resistant *S. aureus* (MRSA). AP-AgNPs exerted a bactericidal mode of action *via* interaction with the cell membrane, leading to its damage and disruption of cell surface structure. Additionally, AP-AgNPs prevented the biofilm development by MRSA through destructing its mature biofilm and disturbing cell adhesion.

AgNPs biosynthesized by *Penicillium aculeatum* showed a higher antimicrobial activity against the tested bacteria and fungi compared to the Ag^+^ ions [75]. The recorded IZD for AgNPs were 12.83 ± 0.58 mm against *S. aureus*, 12.17 ± 1.04 mm against *E. coli*, 16.17 ± 1.04 mm against *B. subtilis*, 15.33 ± 0.76 mm against *P. aeruginosa*, and 18.17 ± 1.26 mm against *C. albicans*. These recorded IZD were considerably higher than those of AgNO_3_, which recorded 11.00 ± 0.50 mm against *S. aureus*, 9.00 ± 1.00 mm against *E. coli*, 12.50 ± 1.50 mm against *B. subtilis*, 13.33 ± 0.58 mm against *P. aeruginosa*, and 14.33 ± 1.04 mm against *C. albicans*, indicating that AgNPs possess a higher antimicrobial efficacy compared to AgNO_3_ [140]. Pereira et al. [141] reported that AgNPs biosynthesized by *P. chrysogenum* MUM displayed a greater biological activity with MIC of 2.89 µg/ml, compared to fluconazole. However, they expressed a lower activity compared to itraconazole, terbinafine, and chemical-AgNPs. Such differences may be attributed to the diversity of NPs’ size and capping. Ilahi et al. [71] demonstrated that AgNPs biosynthesized using cellular extracts of the endophytic fungus *F. oxysporum* NFW16, isolated from *Taxus fauna*, were effectively adsorbed onto bandages and cotton fabrics. These AgNPs exhibited an antibacterial activity against the ATCC isolates in addition to several other clinical isolates. These findings demonstrate that AgNPs can be effectively incorporated onto the cotton fabrics and bandages, suggesting their potential application in preventing severe microbial infections and promoting wound healing. Furthermore, the antibacterial activity displayed by AgNP’s impregnated cotton was higher than the bandage. This difference may be attributed to the lower adsorption capacity of the bandage, as its pore structure may limit AgNP retention compared to cotton, which provides a greater available surface area for nanoparticle adsorption.

### Antifungal potency

In combination with the antifungals, the biosynthesised AgNPs have demonstrated an appreciable antifungal efficacy against all the tested pathogenic fungi. However, such activity has substantially increased in combination with the standard antifungal drugs [142]. The combination of AgNPs synthesized by *Helminthosporium* sp. with ketoconazole caused remarkable inhibitory zones against all tested fungal species, including *Aspergillus* spp., followed by *Fusarium* sp., *Bipolaris* sp., and *Culvularia* sp. Meanwhile, similar results have been observed by AgNPs synthesized using *Helminthosporium* sp. in combination with amphotericin B. However, *Chaetomium* sp. extract-mediated synthesis of AgNPs exhibited a noticeable inhibitory zone against *Culvularia* sp. in combination with amphotericin B, followed by *Bipolaris* sp., *Fusarium* sp., *A. niger*,* A. flavus*, and *A. fumigates.* These results confirm that AgNPs display an appreciable antifungal potency upon combination with antifungals [143].

In the medical sector, pathogenic fungi are known to use several mechanisms to avoid the host immune system, causing negative impacts on human health [69]. The use of nano-sized drugs has overcome the limitations of the existing antifungals in treating the fungal diseases caused by the pathogenic strains. Radhakrishnan et al. [144] have reported the AgNP’s mediated inhibition of the fungal growth. The penetration of biosynthesized AgNP’s into the fungal cell walls may be facilitated by their small size and distinctive surface properties, which promote interactions with and disruption of the fungal cell wall and cellular components. AgNPs exerted several actions, mainly adherence to the fungal cell membrane, generation of the ROS, and interference with the fatty acids, thereby altering the membrane fluidity. Wang et al. [145] reported that AgNPs disrupted the natural architecture of the DNA. Interestingly, AgNPs substituted the hydrogen bonds adjoined to the nitrogen atoms in the purines and pyrimidines, inhibited the fungal cell division, and interacted with the DNA bases forming the crosslinks. BalaKumaran et al. [146] studied the cytotoxic, antifungal, and antioxidant potentials of the *A. terreus*-mediated AgNPs compared to those of AuNPs. The obtained results demonstrated that AgNPs alone exhibited an antifungal potency, whereas AuNPs showed no activity. AgNPs (50 µg/ml) expressed the maximum antifungal efficacy against several pathogens, mainly *Penicillium* sp., recording 60.16%, followed by *A. flavus* (49.91%), and *C. lunata* (47.24%); however, a minimum inhibitory activity was detected against *Colletotrichum* sp., recording 39.98%.

Similarly, in the agricultural sector, a good antifungal activity of the mycosynthesized AgNPs has been reported against many phytopathogenic fungi [147]. Madbouly et al. [148] investigated the *in vitro* and *in vivo* antifungal efficacy of AgNPs mycosynthesized using a culture filtrate of *C. globosum* against the fungal causal agent of Fusarium wilt of tomato (*F. oxysporum* f. sp. *lycopersici*). The obtained results revealed a considerable *in vitro* antifungal potential of AgNPs. Meanwhile, in the greenhouse experiment, the tested AgNPs reduced the wilt disease severity. Moreover, in soil pre-infested with *F. oxysporum*, AgNPs caused a remarkable promotion of several tested growth parameters in the tomato seedlings, including fresh weight of shoot and root, in addition to their height.

### Antiviral efficacy

Chemically synthesized AgNPs have also demonstrated substantial antiviral activity against a range of viruses, including herpes simplex, monkeypox, HIV-1, respiratory syncytial, hepatitis B, and the Tacaribe virus [149]. Sun et al. [150] reported that AgNPs at 5 mM reduced the percentage of apoptotic Hut/CCR5 cells from 49% to 35%. Moreover, AgNPs in the range of 1–10 nm were attached to the HIV-1 virus through undergoing a size-dependent interaction. At the early stage of viral replication, AgNPs exerted an anti-HIV activity, acting as inhibitors of viral entry or virucidal agents. The mycosynthesized AgNPs also showed excellent antiviral activities. AgNPs mycosynthesized by *Aspergillus* spp. expressed an inhibitory activity in a dose dependent manner within the range of 30–210 µg/l against several bacteriophage strains, causing a decrease of the plaque number to 2 plaque forming units (PFU). Moreover, at MNP’s concentrations of 210–240 µg/l, they caused total inhibition of viral growth [151]. AgNPs biosynthesized by *A. ochraceus* reduced the viral plaque from 80 to 32 PFU after half an hour, compared to the control (78 PFU) [152]. At 8–12 µg/l, AgNPs of small size (*i.e.*, 3–10 nm) that have been mycosynthesized by *A. niger* manifested a potent antiviral potential, along with a complete inhibition of viral growth in *E. coli* [153]. At 10 µg/ml, AgNPs biosynthesized by *Curvularia* spp. and *F. oxysporum* displayed an excellent antiviral potency, recording 80–90% growth inhibition of HSV-1 and HPIV-3 viruses [154]. The IC_50_ value of the chemical AgNPs tested against HIV-1 virus ranged from 0.44 to 0.91 mg/ml, and completely inhibited HSV-2 viral replication at 100 µg/ml [155], demonstrating that mycosynthesized AgNPs possess a higher antiviral efficacy compared to the chemical ones.

### Antioxidant activity

Bagur et al. [136] studied the antioxidant potential of biogenic AgNPs and EFE using bio-cellular antioxidant activity and chemical (DPPH) and (ABTS^+^) assays. The scavenging activity of the tested samples of ABTS and DPPH was compared to that of ascorbic acid, which was used as a positive control. Both the EFE and the biosynthesized AgNPs expressed a free radical scavenging potential that is dose-dependent. Moreover, compared to EFE, the AgNP’s DPPH and ABTS radical scavenging activity was more substantially active; however, this activity was close to that of ascorbic acid (at 100 µg/ml). A study conducted by Popli et al. [12] developed an easy and ecofriendly assay for the green synthesis of AgNPs using a water extract of the endophytic fungus *Cladosporium* sp. (CsAgNPs), and investigated the effects of AgNPs as antioxidants, anti-butyrylcholinesterase (BChE), anti-diabetic, and anti-acetylcholinesterase (AChE) agents. The CsAgNPs displayed an ambitious antioxidant efficacy, while the radical scavenging ability showed an increment in a dose-dependent manner. In the ferric ion reducing antioxidant power (FRAP) assay, CsAgNPs displayed an appreciable antioxidant activity compared to the *Cladosporium* sp. extract, which may be attributed to the presence of phytochemicals in the fungal extract. According to Kharat and Mendhulkar [156]; Manjunath et al. [76], the antioxidant potential of AgNPs was attributed to the presence of a phenolic compound. Meanwhile, the tested endophytic aqueous extract exhibited several important phytochemicals, which were reported as strong antioxidants.

### Anti-cancer potential

Given their remarkable potential for nanodrug development, fungi represent promising platforms for the production of novel drugs, antimicrobial agents, and bioactive metabolites. This is attributed to the production of a diverse array of bioactive metabolites (~ 6400) by various fungal species [35]. The Ag-based nanostructures possess several beneficial functions; however, they have limited application in the biomedical field due to their instability and unacceptable toxicity in addition to the oxidation of their surfaces in the presence of an oxygen-containing biofluid [157]. Surface capping of these nanostructures using a biopolymer or a bio-active agent may be used to overcome this problem. Furthermore, capping of AgNPs can reduce the stability against oxidation, undesirable cytotoxicity, and enhance the AgNP’s biocompatibility associated with a better cellular uptake; thus, causing an increase in their therapeutic potentials [158].

Cancer is regarded as one of the leading causes of death worldwide and is therefore considered one of the most fatal diseases [159]. Several large-sized anticancer drugs have faced remarkable shortages in inducing total eradication of the cancer cells, as they are unable to penetrate into the cancer cell’s matrix. Moreover, continuous manipulation of high conventional drug’s doses may cause deleterious impacts in the environment. The effective use of these anticancer drugs is hindered by their stability, penetration, low bioavailability, and solubility [69].

**Fig. 2:**
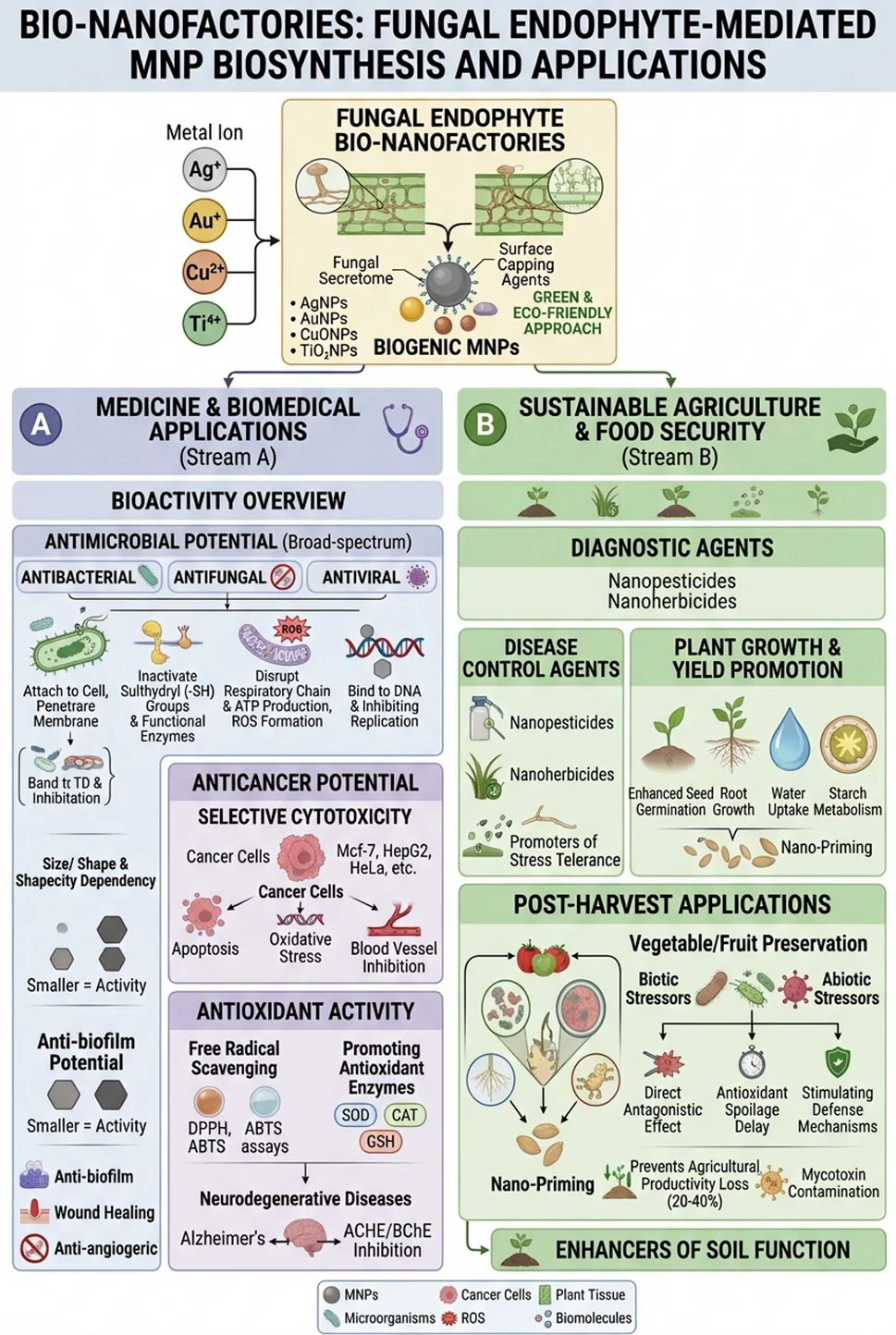
Biosynthetic routes and applications of endophytic fungal-mediated metal nanoparticles in nanomedicine and sustainable agriculture, highlighting their antimicrobial, anticancer, and antioxidant activities, as well as applications in agricultural disease control, nano-priming, and post-harvest food preservation

MNPs have displayed a remarkable bioactivity and promising therapeutic potential in cancer treatment. CuONPs possess medicinal properties and play a vital role in cancer treatment. Radhakrishnan et al. [160] reported the biosynthesis of CuONPs using an enzymatic suspension of two endophytic fungal strains, namely *A. sydowi* and *A. versicolor* isolated from *Azadirachta indica*. CuONPs exhibited high antimycotic, antioxidant, and antibacterial activities at a low concentration. Additionally, using HET-CAM cell lines, the anti-angiogenic potency of the CuONPs in chick embryos led to high inhibition of the blood vessels. The findings of this study revealed the recognizable role of CuONPs in the remedy of cancer. Several biological techniques provide a green and sustainable strategy to biosynthesize CuNPs. CuNPs are becoming ambitious candidates for treatment of cancer due to their unique physicochemical features. They exhibit an anticancer activity *via* inducing apoptosis of the cancer cells, oxidative stress, and DNA damage, ultimately leading them to diminish. Additionally, CuNPs can be functionalized with several other targeting ligands, facilitating their unique binding to the cancer cells, causing an increment in their specificity, and decreasing damage to the healthy cells. Furthermore, CuNPs may exhibit synergistic effects when combined with other anticancer agents, thereby enhancing their therapeutic efficacy in conjunction with conventional treatments such as chemotherapy and radiotherapy [161]. In another study carried out by Danagoudar et al. [162], AgNPs were synthesized using an aqueous extract of the endophytic fungus *A. austroafricanus* CGJ-B3, which showed a considerable cytotoxic, antioxidant, and anti-choline esterase activity. AgNPs expressed an appreciable cytotoxic activity against several cancer cell lines, including MCF-7, A431, and HepG2, compared to the normal cells (HEK-293). These results revealed the possible use of AgNPs as novel therapeutic agents to treat cancer and Alzheimer’s diseases. Sulaiman et al. [163] tested the AgNP’s anti-proliferation activity against three different cancer cell lines, namely HepG2 (hepatoma), MCF-7 (breast cancer), and A431 (skin carcinoma), along with a normal cell line HEK-293 (human embryonic kidney). AgNPs displayed an effective cytotoxic potency against all the tested cancer cell lines. Moreover, the impact of AgNPs on cancer cells helped in predicting the tumor reduction efficacy of these MNPs, in addition to their cytotoxic capability. Lichota and Gwozdzinski [164] reported that AgNPs manifested the least toxicity against the normal cell lines (HEK-293). Their toxicity to both the normal and tumor cells represents the principal disadvantage of most of the used chemical anticancer drugs. According to Hu et al. [142], a drug compound that selectively induces cytotoxicity in tumor cells while exhibiting minimal toxicity towards normal cells would be highly desirable for use as an anticancer therapy. Thus, the mycosynthesized AgNPs using *A. austroafricanus* can be used for treatments of different types of cancer. Endophyte-mediated AgNPs have demonstrated potential anticancer activity by inducing apoptotic pathways in tumor cells. Similarly, titanium dioxide NPs (TiO_2_NPs) have attracted a substantial attention due to their unique physicochemical characteristics and various applications. TiO_2_NPs were biosynthesized using an endophyte, *Aspergillus* sp extract, acting as an ecofriendly stabilizing and reducing agent. *In vitro* assays showed their potent anticancer potential against the MCF7 breast cancer cell line. The findings of this study indicated that TiO_2_NPs possess an anticancer activity, suggesting their binding to the matrix metalloproteinase-3 (MMP3) [165]. BalaKumaran et al. [146] compared the cytotoxic, antioxidant, and antifungal efficacies of AgNPs biosynthesized using *A. terreus* with those of AuNPs. The AgNPs had a size range of 8–20 nm and were spherical in shape, while the AuNPs demonstrated a size range of 10–50 nm with an anisotropic shape. The cytotoxicity of AgNPs and AuNPs was evaluated against six different cancer cell lines and a normal cell line (Vero). The obtained results revealed that AgNPs and AuNPs did not demonstrate a noticeable cytotoxic activity against the normal cell line. However, AgNPs exhibited a remarkable cytotoxic potential against the MCF-7 cells (IC_50_: 2.90 µg/ ml), while AuNPs displayed a maximum cytotoxicity against the HepG2 cells (IC_50_: 25.79 µg/ml). Therefore, the biosynthesized MNPs are highly effective in the treatment of cancer, displaying cytotoxicity towards cancer cells by inducing apoptosis and generating ROS. Collectively, the diverse applications of endophytic fungal-mediated MNPs, including their antimicrobial, anticancer, and antioxidant activities, as well as their potential for agricultural disease control, are summarized in Fig. 2.

### Cytotoxicity of the metal nanoparticles

Silver MNPs exhibit cytotoxic potentials that differed according to the exposure concentration and the type of cell line [75]. AgNPs mycosynthesized using *Pestalotiopsis microspora* demonstrated an appreciable cytotoxic activity against several cell lines, including human ovarian carcinoma, prostate carcinoma, lung adenocarcinoma cells, and mouse melanoma [166]. For the normal human cells, the fungal-derived AgNPs displayed less toxicity compared to the chemical and the green synthesized AgNPs. At a concentration range of 2–4 µg/ml, the MAgNPs and PAgNPs mycosynthesized by *P. microspore* and *A*,* indica*; respectively, did not show a considerable impact on the HaCaT cell’s viability, even after 24 h of incubation, where 98% of the cells stayed viable. In contrast, under similar conditions, treatment with 4 µg/mL of chemically synthesized AgNPs for 24 h exerted greater cytotoxic effects on healthy cells, resulting in less than 60% viability of HaCaT cells [167]. The kidneys and liver are the primary human organs associated with the accumulation of Ag⁺ ions following AgNP exposure, regardless of the route of administration, including the nasal route [168], intravenously [169], or orally [170]. AgNPs derived from various sources possess different impacts on the health of mammals. Multiple *in vivo* studies conducted on rats treated with 10 mg/kg AgNPs green synthesized using *Ficus religiosa* leaf extract showed the accumulation of Ag^+^ ions in the rat’s lungs, liver, and brain, with corresponding concentrations of 4.77, 3.94, and 3.043 µg/g tissue, respectively [171]. The cytotoxicity of AgNPs depends on their surface area, size, attachment efficiency, and internalization. AgNPs with a small size are able to internalize into the mammalian cells and cause cytotoxicity through nucleus damage, activation of apoptosis or mitochondrial pathways, and ROS generation [172]. In the previous study conducted by Sivalingam [173], AgNPs were green synthesized using *Swietenia macrophylla* leaf extract. The SM-AgNPs displayed a considerable cytotoxicity towards the MCF-7 breast cancer cells. The recorded IC_50_ value revealed that the SM-AgNPs outperformed the conventional AgNPs and the plant extract. In another study conducted by Hu et al. [142], the effective cytotoxicity of *Tradescantia pallida* (Tp-AgNPs) was attributed to their small size of < 50 nm, reflecting both a cytotoxicity and an excellent anticancerous potential. The AgNP’s cytotoxicity is affected by several factors, such as shape, size, composition, surface charge, and capping molecules [174]. The definite mechanisms of MNPs cytotoxicity have not been completely proven; however, several proposed hypotheses are listed below:

1. AgNPs generate a continuous flux of Ag^+^‏ ions, which can affect cells through incorporation into cellular matrices or binding to cellular substrates. Ag^+^‏ ions may bind to the sensitive cellular targets or be transported to the cell membrane, where they can penetrate into the cells through endocytosis and/or attachment [175]. A previous study reported that most of Ag^+^ ions originate from the dissolution or oxidation of the AgNPs, which typically become mediated by the protons and/or the other components or oxidized by the dissolved O_2_ [167].

2. The AgNPs are internalized by cells through endocytosis, where they can induce oxidative stress [177]. AgNPs bind to the thiol groups of the enzymes, including the NADH dehydrogenase, and generate ROS by disrupting the respiratory chain. These ROS may induce several deleterious metabolic processes, DNA damage, lipid peroxidation, biomarker production, protein modification, and antioxidant enzymes [167]. Furthermore, the cellular uptake of AgNPs occurs primarily through endocytosis, followed by their trafficking from endosomes to lysosomes, where they are exposed to an acidic condition with a pH range of 4–5 [179]. The release of Ag⁺ ions from AgNPs increases under acidic conditions, which may provide useful insights into the mechanisms underlying AgNPs toxicity.

3. AgNPs may bind to the cell membrane and use an electrostatic attraction to induce ROS. These ROS influence the integrity of the membrane and cause cell death [180]. Treatment with AgNPs may promote the expression of several oxidative stress-related enzymes, including CAT, glutathione peroxidase 1 (GP×1), and SOD1 [181].

4. In aqueous environments, the toxicity induced by AgNPs is affected by several parameters, including ionic strength, pH, and temperature [182]. Additionally, the cytotoxicity is also influenced by the properties of the AgNPs, mainly the coating materials used in their synthesis and their surface charge. However, there is emphasized evidence that AgNPs with small size may display higher cytotoxicity [183]. Table 1 summarizes selected representative studies from the literature on fungal-mediated synthesis of metal and metal oxide NPs.

**Table 1:** Comparative overview of endophytic fungal-mediated metal nanoparticle biosynthesis and their biomedical and agricultural applications

Fungal species/strain	MNP type	Synthesis route	Particle size (nm)	Biomedical activity	Agricultural activity	Toxicity/safety	Refs.
*Cladosporium sphaerospermum* F16 endophyte isolated from tomato	AgNPs	Extracellular (cell-free fungal extract)	15.1 ± 1.0	No biomedical activity experimentally evaluated in this study.	Not reported	No direct toxicity/safety assay reported.	[60]
*Chaetomium globosum* SQ3, endophyte isolated from *Panax notoginseng*	AgNPs	Extracellular (cell-free fungal filtrate)	2.9–31.34	Antioxidant, antifungal against *Phytophthora infestans*, *Botrytis cinerea*,* Helminthosporium maydis*. Antibacterial against *S. aureus*,* E. coli*, and *Bacillus subtilis.*	Reduced *B. cinerea* lesion diameter on cherry tomato and reduced decay rate. They also reduced weight loss and helped preserve firmness, and antioxidant status.	No direct human/animal toxicity or cytotoxicity assay was reported.	[61]
*Fusarium oxysporum*, *F. avenaceum*, *F. falciforme* isolated from *Salvia persepolitana*	AgNPs	Extracellular (fungal cell filtrate)	50–70	Dose-dependent antibacterial activity against *S. aureus* and *E. coli.*	Not reported	Not reported	[62]
*Penicillium oxalicum*, endophyte associated with *Amoora rohituka*	AgNPs	Extracellular (aqueous fungal extract)	5–23	Antibacterial activity against *E. coli* and *S. aureus*. Antifungal activity against *Aspergillus niger*,* A. flavus*,* A. luchuensis* and *Candida albicans*; antioxidant, anticancer.	Not reported	Not reported	[69]
*F. oxysporum* NFW16, endophytic fungus isolated from *Taxus fauna*	AgNPs	Extracellular; fungal cell filtrate (CF).	30–36.1	Strong in vitro antibacterial activity against Gram-positive and Gram-negative pathogens against MRSA and *P. aeruginosa*, *E. coli*. AgNP-impregnated cotton and bandage were also antibacterial; cotton gave maximum ZOI of 44 mm against *S. aureus* and *E. coli*.	Potential biomedical application as an antimicrobial agent and antimicrobial textile/wound-dressing material.	No dedicated cytotoxicity assay was reported.	[71]
*P. citrinum* (marine endophytic fungus isolated from *Sargassum wightii*)	AuNPs	Extracellular (aqueous extract of fungal biomass/filtrat)	60–80	Antioxidant activity demonstrated by DPPH radical-scavenging assay.	Not reported	No direct toxicity/safety assay was reported.	[76]
*A. brasiliensis* (sugarcane endophytic fungus)	AgNPs	Extracellular; aqueous endophytic fungal extract, co-stabilized with carboxymethylcellulose	3.5 ± 1.0	Antibacterial activity against *S. aureus* and *S. epidermidis*.	Not reported	No direct mammalian cytotoxicity or safety assay was reported.	[79]
*Alternaria* sp. (endophytic fungus isolated from *Raphanus sativus*)	AgNPs	Extracellular; mycelium-free hot-percolation biomass filtrate/supernatant	4–30	Strong antibacterial activity against methicillin-resistant *B. subtilis*,* S. aureus*,* E. coli* and *Serratia marcescens*.	No direct plant-growth, crop-protection or field-efficacy experiment was conducted.	No direct mammalian cytotoxicity/safety assay was reported.	[83]
*Calonectria eucalypti* HECL10 (endophytic fungus isolated from *Saxifraga stolonifera*)	AgNPs	Extracellular; aqueous extract of washed fungal mycelium	0.84–17.1	Antimicrobial activity against *E. coli*,* P. aeruginosa*,* S. aureus* and *C. albicans*; significant anticancer activity against A549, A375, HeLa and HepG2 cells; strong antioxidant activity against DPPH, ABTS and OH.	Not reported.	Good in vitro biocompatibility toward non-cancerous HaCaT cells: 6.25–25 µg/mL caused no significant reduction in cell survival.	[115]
*A. clavatus* AzS-275 (endophytic fungus isolated from *Azadirachta indica*)	AgNPs	Extracellular; fungal system challenged with 1 mM AgNO₃	10–25	Antimicrobial activity against *C. albicans*, *Pseudomonas fluorescens* and *E. coli*.	Not reported	No dedicated mammalian cytotoxicity or in vivo safety assessment was reported in the article.	[138]
*A. parasiticus* EA-1 (RCMB 150) (endophytic fungus isolated from *Reseda arabica*)	AgNPs	Extracellular; ethyl acetate extract of the fungal culture/filtrate	5.3–72.1	Strong anti-MRSA activity; AP-AgNPs also inhibited MRSA biofilm formation and disrupted established biofilms. In a mouse model of MRSA superficial skin infection.	Not reported.	These results indicate limited hemolytic activity at the tested concentration, but they do not constitute a complete toxicity/safety assessment.	[139]
*Talaromyces purpureogenus* (endophytic fungus isolated from *Pinus densiflora*)	AgNPs	Extracellular; cell-free mycelial extract (MEEF)	25–50	Antibacterial activity against Gram +ve and Gram -ve pathogens; anti-proliferative activity against human lung carcinoma A549 cells; In-vitro cell wound-healing activity in NIH3T3 cells.	Not reported	Tp-AgNPs were reported as not cytotoxic to normal NIH3T3 cells. The study also evaluated cytotoxicity in A549 cancer cells.	[142]
*C. globosum* KSA-EGY-19 (isolated from corn)	AgNPs	Extracellular; cell-free fungal extract/filtrate	11–14	No biomedical activity was evaluated.	In vitro inhibition of *F. oxysporum* growth; Wilt severity was reduced by 90%.	No direct mammalian toxicity/safety assay was reported in this study.	[148]
*Alternaria* sp. (isolated from *Dryopteris* spp.)	AgNPs	Extracellular; cell filtrate	7–20	Low/inefficient antiviral activity against HSV-1, HSV-2 and HPIV-3 limiting antiviral use.	Not reported	Cytotoxic effects in Vero cells after 10 h at 5–10 µg/mL.	[154]
*F. oxysporum* (isolated from *Musa paradisiaca*)			4–13	Strong antiviral activity against HSV-1 and HPIV-3; dose-dependent inhibition of HSV-1, HSV-2 and HPIV-3 in post-treatment/virus-preincubation assays.		Very minor cytotoxicity in Vero cells; no decrease in cell viability up to 50 µg/mL was observed under the tested conditions.	
*Curvularia* sp. (isolated from *Catharanthus roseus*)			5–23	Antiviral activity against HSV-1, HSV-2 and HPIV-3; and dose-dependent inhibition in post-treatment and virus-preincubation assays. Activity against HPIV-3 was weak.		Very minor cytotoxicity in Vero cells; reported as having relatively low toxicity compared with Alternaria and Phoma-derived AgNPs.	
*C. indicum* (isolated from *Selaginella bryopteris*)			10–31	Strongest antiviral activity against HPIV-3, reaching ~ 90% inhibition; also showed strong, dose-dependent inhibition of HSV-1, HSV-2 and HPIV-3.		Very minor cytotoxicity in Vero cells; no decrease in cell viability up to 50 µg/mL was observed.	
*Phoma* sp. (isolated from *Syzygium cumini*)			7–20	Low/inefficient antiviral activity against HSV-1, HSV-2 and HPIV-3.		Toxic to Vero cells after; residual toxicity interfered with interpretation of some antiviral assays.	
*A. versicolor* FCPRS11 (endophytic fungus isolated from *Azadirachta indica*)	CuONPs	Extracellular; fungal extracellular enzymes induced by 0.1 mM CuSO4	76	Antibacterial and antifungal activity against the tested pathogens; antioxidant activity through DPPH, nitric oxide radical scavenging and reducing power; anti-angiogenic activity in HET-CAM.	No direct plant-growth, crop-protection or field-efficacy experiment was conducted.	No direct mammalian toxicity/safety assay was reported.	[160]
*A. austroafricanus* CGJ-B3 (endophytic fungus)	AgNPs	Extracellular; endophytic fungal-mediated green synthesis	2–51	Antioxidant, cytotoxic and anti-cholinesterase activity; cytotoxicity against MCF-7, A431 and HepG2 cancer cells than HEK-293 noncancerous cells; potential therapeutic application against cancer and Alzheimer’s disease.	Not reported	Cytotoxicity was assessed using XTT; AgNPs showed greater cytotoxic activity against cancer cell lines than HEK-293 cells.	[162]
*Aspergillus* sp. AT22 (endophytic fungus; OR327425)	TiO₂ NPs	Extracellular; fungal culture filtrate	1.4–20	Weak antibacterial activity against *S. aureus* and *E. coli*. Anticancer/cytotoxic activity against MCF7 and HepG-2 cells.	Not reported	ADME prediction indicated potential safety concerns: mutagenicity 0.99, carcinogenicity 0.85, drug-induced liver injury 0.88, and skin reaction.	[165]
*Pestalotiopsis microspora* VJ1/VS1 (KX213894; endophytic fungus isolated from *Gymnema sylvestre*)	AgNPs	Extracellular; aqueous fungal culture filtrate	2–10	Effective antioxidant activity against DPPH and H₂O₂ radicals; significant cytotoxicity against B16F10, SKOV3, A549 and PC3 cancer cells; antimicrobial activity against bacterial and fungal pathogens.	Not reported.	Biocompatible toward normal CHO cells. No adverse effect at the tested concentrations in normal cells, while cancer cells showed concentration-dependent apoptotic changes.	[166]
*A. flavipes* AUMC 15,772 (endophytic fungus isolated from *Lycium shawii*)	AgNPs	Extracellular; mycelial filtrate	2.8–17.7	Strong antibacterial activity against multidrug-resistant wound pathogens *P. aeruginosa* PA-09, *K. pneumoniae* KP-1, *E. coli* EC-3 and *S. aureus* SA-17.	Not reported	High in vitro biocompatibility with normal human skin fibroblasts; No significant toxicity. No in vivo safety assessment was reported.	[185]
*A. terreus* BR.1 (endophytic fungus isolated from *Allium sativum*)	CuO-NPs	Extracellular; cell-free fungal biomass filtrate/metabolites	15–55	Antibacterial (*B. subtilis*,* P. aeruginosa*,* E. coli* and *C. parapsilosis*), and anti-Candida (*C. albicans*,* C. glabrata* and *C. tropicalis*). Anticancer activity against MCF7 and PC3 cells. DPPH antioxidant activity.	Not reported	In vitro biocompatibility toward normal Vero and Wi38 cells was demonstrated. No in vivo toxicity or environmental safety assessment was reported.	[186]
*A. fructus* (marine endophytic fungus; seaweed-associated)	AgNPs	Extracellular; cell-free filtrate (CFF) from hypo-osmotic/distilled-water culture	40.64	Not reported	Not reported	No direct mammalian, environmental, or ecotoxicity assay reported.	[187]
*Emericellopsis maritima* (marine endophytic fungus; seaweed-associated)	AgNPs	Extracellular; cell-free filtrate (CFF) from hypo-osmotic/distilled-water culture	25				
*P. toxicarium* (marine endophytic fungus; seaweed-associated)			Not reported				
*Purpureocillium lilacinum* (marine endophytic fungus; seaweed-associated)			191.60				
*T. pinophilus* (marine endophytic fungus; seaweed-associated)			Not reported				
*P. oxalicum* (endophytic fungus isolated from *Tecomella undulata*)	FeNPs	Extracellular; fungal mycelial extract/filtrate	140–170	Environmental-remediation activity: FeNPs effectively decolorized methylene blue in the presence of H₂O₂,	Not reported	No mammalian cytotoxicity, ecotoxicity, or in vivo safety assessment was reported.	[188]
*Phomopsis liquidambaris* SA1 (endophytic fungus isolated from *Salacia chinensis*)	AgNPs	Extracellular; cell-free fungal supernatant/filtrate	18.7	Strong dose-dependent antibacterial activity against *Vibrio cholera*, *Salmonella typhi*, *Micrococcus luteus*, *Proteus mirabilis*, *E. coli*, *Klebsiella pneumonia*, *Shigella flexneri* and *P. putida*; larvicidal activity against *Aedes aegypti* and *Culex quinquefasciatus*.	Larvicidal/mosquitocidal activity: dose-dependent mortality of II and IV instar *A. aegypti* and *C. quinquefasciatus*.	Very low/acceptable hemolytic activity in vitro; reported as biocompatible and suitable for biomedical applications.	[189]
*F. chlamydosporum* MW341592.1 (endophytic fungus isolated from *Eucalyptus sideroxylon*)	ZnO NPs	Extracellular; cell-free fungal filtrate	19.3	Antibacterial activity against multidrug-resistant *E. coli* and *P. aeruginosa;* (anticancer activity against HCT-116 and CACO2 cells).	Not reported	Cytotoxicity was demonstrated in cancer cell lines in a concentration-dependent manner; no dedicated normal-cell safety study was reported.	[190]
*F. chlamydosporum* MW341592.1 (endophytic fungus isolated from *Eucalyptus sideroxylon*)	AuNPs	Extracellular; cell-free fungal filtrate	22.1				

“Not reported” indicates that the information was not reported in the supplied full-text article

### Synergistic impacts of the metallic nanoparticles

Worldwide, the incidence of human infections caused by multidrug-resistant (MDR) bacteria, as well as by untreated bacterial infections, is continuously increasing, highlighting the widespread prevalence of antibiotic-resistant bacteria [190]. MNPs, including Au, Ag, Fe_3_O_4_, CuO, ZnO, and TiO_2_, are commonly used as antimicrobial agents [191]. The high surface-to-volume ratio and nanoscale size of MNPs are major factors influencing their antimicrobial properties, leading to strong interaction with the microbial membranes. This causes disruption and damage to internal cellular structures, penetration into cells, and finally cause microbial cell death [134]. MNPs show a broad-spectrum antimicrobial activity due to non-specific interactions between released metal ions and microbial biomolecules [192]. Following their release into the extracellular environment, metal ions enter microbial cells and disrupt essential cellular processes. Inside the microbial cell, the metal ions affect the cellular structure and elicit the production of ROS, leading to disrupting the functions of the cell. These are often attributed to the formation of strong coordination bonds with the oxygen, sulfur, and nitrogen atoms existing in the organic compounds and the biomolecules [193]. Compared to the antimicrobials alone, the combinations of antimicrobials and MNPs have exhibited a superior potential against the microbial resistance [194]. This may lead to decreasing the duration and the dose requirements of the antimicrobial treatments and reduce the development of antimicrobial resilience [195]. MNPs can be classified into several types based on the composition of their constituent components:

#### Monometallic nanoparticles

Due to their distinct mechanisms of action compared to conventional antibiotics, MNPs are among the most widely studied inorganic NPs, offering promising alternatives for combating MDR bacteria. They target several biomolecules in the bacterial cell, impeding the development of resistant strains [196]. AuNPs and AgNPs are the leaders among the MNPs.

AgNPs possess an exceptional antimicrobial potency against a wide spectrum of pathogens [197] *via* three main mechanisms of action: (1) They interact and penetrate into the microbial cell membrane [198], leading to membrane lipid peroxidation and protein inactivation, followed by disrupting the membrane integrity, dysfunction of transport proteins, and leakage of cellular contents [199]. (2) AgNPs induce damage to the intrinsic structures, eliciting the generation of ROS, causing redox hemostasis disruption, affecting signal transduction pathways, and Na^+^/K^+^ ATPase pump [200]. Cell death may result from the interaction of the MNPs with the DNA, leading to DNA damage and subsequent protein inactivation [201]. 3) The release of Ag^+^ ions [202] in consistency with the other two mechanisms may cause their interaction with the cell components, affecting the genetic material and the metabolic pathways, mainly due to their size and charge [203].

Due to their low toxicity, biocompatibility, controlled synthesis, inertness, and simplicity, compared to the other nanomaterials, AuNPs are regarded as one of the most important types of MNPs. Similar to AgNPs, AuNPs cause the intracellular components leakage through disrupting the structure and integrity of the cell membrane [204]. This mode of action is similar to the apoptosis-like cell death, where AuNPs induce cell membrane depolarization, a subsequent increase in Ca^+ 2^ ions concentration in the cytoplasm, and induction of fragmentation of DNA inside the bacterial cell, finally causing an apoptosis-like death [205]. Furthermore, the membrane potential becomes changed and the ATP synthase activity is reduced, causing a metabolic dysfunction [206], in addition to a recognizable increment in the levels of intracellular ROS [207].

The antimicrobial activity of MNPs can be modulated by combining them with suitable compounds, such as transition metals. The antimicrobial potential of CdS combined with Co and Fe and ZnONPs combined with Fe was evaluated in a previous study. The results showed that ZnONPs combined with Fe expressed a reduced antibacterial potency against the tested Gram-negative bacteria, due to a decrease in the positive charge existing on the surface of MNPs and an alteration in the surface morphology of the ZnONPs. However, CdSNPs combined with Fe showed an increment in their antibacterial potential, but no recognizable alteration was observed in the activity of CdSNPs combined with Co [208]. Alterations in the size and surface charge of MNPs are considered key factors influencing the antimicrobial efficacy observed following their combination with transition metals.

#### Metallic nanoparticles combined with antibiotics

MNPs can overcome multiple bacterial resistance mechanisms through their combination with antibiotics, exhibiting synergistic antimicrobial effects against MDR bacteria, including both planktonic cells and biofilm-forming bacteria [209]. The benefits of this combination are an increment in the bactericidal or fungicidal potency due to a synergistic effect. This leads to a faster antimicrobial activity and an increment in the antimicrobial agent’s penetration into the microbial cells and tissues, reducing the possibility of development of resistant microorganisms and expressing an antimicrobial activity against the pathogens forming biofilms [210]. Furthermore, MNPs do not have a sufficient potential to induce microbial resistance [211]. This is because the action of MNPs takes places *via* direct contact with cell wall of the pathogen irrespective of the necessity for penetrating the microbial cells. This is in addition to the use of particular pathogens’ targets, which may be modified through the response of the microorganism to the presence of these MNPs [212]. Through combination with. MNPs, increasing the efficacy of antibiotics makes it possible to reduce the drug concentration administered to the patient, decrease the systemic toxicity, and shorten the duration of the antibiotic treatment [213]. As an example, synergism of AgNPs combined with the imipenem antibiotic was investigated against a pandrug-resistant *Acinetobacter baumannii*. The results revealed a positive synergistic activity, which has led to more than a fourfold reduction in the AgNP’s MIC values and the imipenem. This may be attributed to the small size of AgNPs, which facilitates their penetration through the cell membrane, disrupts membrane integrity, and enhances the passage of antimicrobial drugs into the cell, resulting in the expression of the MNPs antimicrobial effect at the target site of the microbial cell [214]. Furthermore, regarding the MNP’s antifungal activity, at 100 µg/ml, AgNPs green-synthesized using agro-waste materials and strawberry leaf prevented spores germination of *B. cinerea*, due to an increment in the solution density, leading to cohesion and/or sticking of the fungal hyphae [215].

#### Multi-metallic nanoparticles

Multi-metallic MNPs are defined as those composed of two different metals at least. An enhanced catalytic action is anticipated from the joint action of different metallic oxides and metals existing in a chemical transformation [216]. Compared to the characteristics of the individual components, a synergistic antimicrobial potential of the developed compound may be obtained through the use of a combination of various metal compounds. The AgNPs antimicrobial activity is often attributed to penetration and anchoring of MNPs into the bacterial cell wall, leading to the generation of intracellular oxidative stress and the formation of free radicals inside the cell, causing cell death [217]. On the contrary, inside the bacterial cell, Fe may interact with the amino acids present in the cell wall, mainly the -SH groups of cysteine, mostly susceptible to the electron capture elicited by the oxidative species [218]. Padilla- Cruz et al. [219] reported that by combination of the bimetallic AgNPs and FeNPs, the mode of action of such synergy involves the SH- side chain’s (existing in the cysteine amino acid) oxidation, leading to alterations in the structure of protein, an increased permeability of the cell wall of bacteria, and finally inducing cell death. An increment in the influx of bimetallic MNPs into the cell is often recorded, due to an increased bacterial cell wall’s permeability. An oxidative stress is often initiated through the Ag^+^ ions released inside the cytoplasm, leading to alterations in the DNA and cell membrane morphology disruption. Accordingly, the observed synergy between Fe and Ag induces the cell structure’s destruction and the intracellular biological function’s disruption, causing death of the cell. Most bacterial infections are associated with the formation of biofilms. Compared to the planktonic cells, the structure of these biofilms shows 10–1000 times more resistance to the antibiotics [220]. Accordingly, there is an urgent need to develop new bactericidal agents capable of combating biofilm-associated infections. Ranpariya et al. [221] reported the synthesis of Ag-platinum (Pt) nanohybrids that remarkably inhibited the bacterial biofilm formation. Moreover, they expressed a considerable antimicrobial. synergy upon combination with many antibiotics, including chloramphenicol, ampicillin, streptomycin, and rifampicin against multiple bacterial strains, including *S. aureus*,* P. aeruginosa*, and *E. coli*. The multi-metallic MNPs may be used as an effective tool to treat the bacterial infections due to their wide spectrum of modes of action and the versatility of these modes against the MDR strains.

#### Metallic nanoparticles carry molecules having antimicrobial efficacy

MNPs possess great potential in medicine through acting as carriers of several small biomolecules, including proteins, enzymes, and genes, in addition to drugs [222]. An increment in permeability of microbial cell or cell envelope’s weakening may enhance the potential of some antibiotics; accordingly, the combination between MNPs and the antibiotics may express better efficacy, mostly. through alleviating the side effects often observed with the individual drugs [223]. 

Enhanced ability of the MNPs to penetrate the biological membranes may cause a strong antimicrobial potency, especially upon their functionalization with the antimicrobial agents. High-polar environments often observed inside the bacterial membranes may impede the activity of the hydrophobic antimicrobials through restricting their cell penetration [224]. MNPs may interact with the membrane of the bacterial cell, inducing a modification in the bacterial integrity and the membrane potential *via* hydrophobic, electrostatic, and receptor ligand interactions [225]. Loading the surface of MNPs with a high dose of an antimicrobial agent may increase the delivered effective dose of this antimicrobial, inducing an increment in the biological membrane’s permeability and a subsequent higher uptake by the cell [226]. Due to its increased porosity, the antibiotic molecules combined with MNPs may facilitate simple access to bacterial cell. 

Furthermore, an improvement in MNPs- antimicrobial agent’s stability may be caused by functionalization of MNPs. Under various abiotic stresses, including room temperature, UV exposure, and heat stress, AuNPs conjugated with streptomycin, kanamycin, or ampicillin often exhibit greater antimicrobial stability and activity than their unconjugated counterparts [227]. This conjugation imparts improvements of the pharmacokinetic characteristics of the drug, mainly through a stimulus-controlled and prolonged drug release, prolonged drug half-life, an increment in the poorly soluble drug’s solubility, and systemic circulation time. These effects reduce the toxicity of the antimicrobial drug through lower dosage and drug frequency [228]. Additionally, compared to the traditional therapy, using MNPs as carriers for drug delivery may enable a reduction in the adverse effects of this drug and improve its efficacy. Furthermore, compared to the antibiotic in a free form, conjugation of AuNPs with ciprofloxacin led to a lower hemolytic activity of this combination, thereby alleviating the antibiotic toxicity [229]. A therapeutic system that targets treatment to control the microbial infection and help for rapid identification of the pathogens can be possibly obtained by functionalizing the MNPs with the antibodies. An ambitious treatment strategy that can be used alone or in addition to the existing traditional antibiotic therapy was obtained by functionalizing AuNPs and AgNPs with the antibodies specific for *S. aureus* peptidoglycan to gain a complete eradication of this pathogen; thus, achieving a selective and an extended bacterial death [230]. In agreement with Naseri et al. [231]; Dabbeta et al. [232], additional future studies are highly recommended, such as standardization of MNPs for industrial applications, optimization of the MNP’s biosynthesis reaction conditions, and the development of integrated strategies that combine the physical and chemical synthesis approaches with the sustainability of green synthesis.

The key findings of this study involve the usefulness of manipulating the fungal endophytes as highly efficient bio-nanofactories for the production of potent uniform, stable, and bio-applicable MNPs, and offering a cost-effective and an ecofriendly alternative to the traditional chemical strategies.

The potential novelty of this study lies in the integration of three biological resources, mainly plant, endophytic fungus, and SMs, resulting in the production of MNPs with an enhanced bio-activity. The current review demonstrates that the MNP’s inherit are influenced by the bioactive chemistry of their endophytic fungi. Moreover, it compares a multifunctional bioactivity profile of the same mycosynthesized MNPs. The mycofabricated MNPs show a superior activity compared to the fungal extracts themselves. The current work establishes a relationship between MNP’s characteristics and their bioactivity. Unlike conventional MNP’s synthesis strategies, this approach manipulates the enzymatic and SMs repertoire of endophytic fungi as both reducing and capping agents, potentially imparting unique surface chemistry and enhanced biological characteristics to MNPs.

The main limitations of this study are that many reported bio-activities of the mycosynthesized MNPs, including antibacterial, antifungal, antioxidant, anticancer, etc., are demonstrated *in vitro*. These results do not necessarily translate into efficacy in animals, plants, or humans. This review reports that MNPs possess multiple biological activities without adequately explaining how these activities occur at the molecular level. Long-term environmental consequences, including effects on soil microorganisms, aquatic organisms, bio-accumulation, and trophic transfer, are insufficiently characterized. Laboratory-scale synthesis does not mean that the process can be economically scaled up.

## Conclusion

Recently, endophytic fungi have attracted considerable attention worldwide, as they possess many beneficial features, enabling their use in biosynthesis of metal nanoparticles. Mycosynthesis of nanoparticles using filamentous fungi such as *Aspergillus* spp. and *Fusarium* spp. presents numerous advantages over the other microorganisms and/or plant extracts. Moreover, the mycosynthesis of metal nanoparticles has been shown to be more ecofriendly and cost-effective than conventional chemical and physical synthesis methods. Currently, mycofabricated nanoparticles are used in medicine and agriculture as antimicrobial agents, antioxidants, anticancerous agents, nanopesticides, nanofungicides, nanoherbicides, nanofertilizers, promoters of plant growth, and enhancers of soil function, along with inducing plant stress tolerance. Moreover, metal nanoparticles possess a potent cytotoxicity against cancer cell lines; however, the widespread application of nanoparticles in cancer treatment requires further *in vivo* evaluation, as their small size may also contribute to cytotoxic effects against normal cells. The combination of MNPs with antimicrobials shows a superior efficacy against the multidrug-resistant pathogens compared to the antimicrobials alone. This review emphasizes that metal nanoparticles possess promising bio-applications that benefit the human and represent a potent ecofriendly alternative to their chemical counterparts. Finally, extensive future *in vivo* and field studies on metal nanoparticles are highly recommended before their manipulation as nanodrugs and/or a control strategy for crop diseases in the field.

## Data Availability

No datasets were generated or analysed during the current study.
